# Specimen Menus—Good and Bad

**Published:** 1911-11-25

**Authors:** 


### Specimen Menus—Good and Bad.

THE following remarks will point out why one meal is well-balanced and the other is not. The menus are merely suggestions, one starchy food can be substituted for another, or different vegetables may take the place of those mentioned.

**BREAKFAST (GOOD).**
**Eggs, lightly boiled. Toast. Stewed apples. Coffee or tea with hot milk.**

A well-arranged menu, simple and digestible. The fruit juices aid digestion by stimulating the flow of the gastric secretions. Eggs, only cooked sufficiently to render the albumen they contain soft and jelly-like, afford a valuable food containing every necessary element of nutrition. Coffee has a distinctly stimulating effect on the circulatory and nervous systems, and is therefore a helpful morning beverage for those who can take it. It is most wholesome if taken with plenty of hot milk and but slightly sugared, and the same may be said of tea.

**BREAKFAST (BAD).**

**Porridge with sugar and cream or syrup. Fried eggs and bacon. Rolls. Cocoa.**

Too heavy to be wholesome. The digestive organs are rarely sufficiently active in the morning to be able to deal with such fare in combination. Oatmeal, being rich in fat, should be eaten with milk instead of cream, and with salt in place of sugar or syrup, as these supply too much carbonaceous food. Frying renders eggs extremely difficult of digestion. Morning rolls are usually hot, and hot bread makes any work doubly laborious. Cocoa contains so much nutriment that if it is used the eggs should be omitted.

**DINNER (GOOD).**

**Clear meat soup or light broth. Stewed meat with vegetables. Macaroni and tomatoes. Apple and sago mould.**

A well-planned meal. A thin soup stimulates without appeasing the appetite. Stewing is one of the most wholesome and economical methods of cooking meats, and the alternative dish (macaroni) is a good substitute for animal food. There is no complicated sweet to retard digestion, but a wholesome, simple dish of fruit and an easily-digested starchy preparation.

**DINNER (BAD).**
**Lentil soup. Roast beef Yorkshire pudding. Potatoes. Greens. Stewed bananas and custard. Milk.**

Lentils, meat, eggs, and milk provide far too rich an allowance of nitrogenous matter. Such a meal heavily taxes the digestive organs, and is sure to be followed by mental and physical lassitude, or, if partaken of in the evening, by a restless night. Bananas have such a high food value that they are not suitable for consumption at the same meal as meat. For the same reason milk is out of place, though these two foods by themselves form an excellent light repast.

**SUPPER (GOOD).**
**Thick vegetable soup. Croutons. Hot milk. Savoury omelet. Wholemeal bread and butter.**

Light and nourishing, conducive to a good night's rest. A vegetable soup such as potato, celery, onion, etc., is excellent for supper with the addition of milk, or, if possible, cream to it. The fat necessary to health and lacking in vegetables is thus supplied, and the croûtons (bread fried in dripping) served with it answer the same purpose. Omelets are not only appetising, nourishing, and digestible, but easily and quickly prepared, and if eaten with good sweet bread and butter make a perfect meal. Hot milk is an excellent restorative for those who are tired, and aids, not hinders, slumber.

**SUPPER (BAD).**

**Cold meat. Pickles. Cheese. Bread and butter. Tea or bottled lemonade.**

An undesirable combination for an evening meal, causing the digestive organs to work far into the night. Pickles, if frequently eaten, injure the digestion, and hence the complexion, while cheese is too concentrated a food for supper unless cooked in some such form as a cheese pudding, fondu, or omelet. Tea is the declared enemy of sleep, and should never be taken with a meat meal, while pure water, or barley water, would prove a more wholesome and thirst-quenching beverage than gaseous lemonade innocent of one drop of genuine lemon-juice.

